# Transplantation of *schistosome *sporocysts between host snails: A video guide

**DOI:** 10.12688/wellcomeopenres.13488.1

**Published:** 2018-01-09

**Authors:** Gabriel Mouahid, Anne Rognon, Ronaldo de Carvalho Augusto, Patrick Driguez, Kathy Geyer, Shannon Karinshak, Nelia Luviano, Victoria Mann, Thomas Quack, Kate Rawlinson, George Wendt, Christoph Grunau, Hélène Moné

**Affiliations:** 1 Laboratoire Interactions Hôtes-Pathogènes-Environnements (IHPE), UMR 5244 CNRS/UPVD/IFREMER/UM, University of Perpignan Via Domitia, 58 Avenue Paul Alduy, Bât R, F-66860 Perpignan Cedex, France; 2 Wellcome Trust Sanger Institute, Hinxton, Cambridgeshire, UK; 3 Institute of Biological, Environmental and Rural Sciences , Aberystwyth University, Aberystwyth, UK; 4 Department of Microbiology, Immunology & Tropical Medicine, George Washington University Medical Center, Washington, DC, USA; 5 Institut für Parasitologie, Justus-Liebig-Universität Gießen, Gießen, Germany; 6 Department of Zoology, University of Cambridge, Downing St, Cambridge, UK; 7 Department of Pharmacology, UT Southwestern Medical Center, Dallas, TX, USA

**Keywords:** Schistosomiasis, sporocyst transfer, Biomphalaria, video instruction

## Abstract

Schistosomiasis is an important parasitic disease, touching roughly 200 million people worldwide. The causative agents are different *Schistosoma* species. Schistosomes have a complex life cycle, with a freshwater snail as intermediate host. After infection, sporocysts develop inside the snail host and give rise to human dwelling larvae. We present here a detailed step-by-step video instruction in English, French, Spanish and Portuguese that shows how these sporocysts can be manipulated and transferred from one snail to another. This procedure provides a technical basis for different types of *ex vivo* modifications, such as those used in functional genomics studies.

## Introduction

Schistosomiasis is an acute and chronic parasitic disease caused by blood fluke trematodes of the genus *Schistosoma*. Estimates show that at least 206.5 million people required preventive treatment in 2016 ( WHO, 2017). This disease is spread throughout tropical and subtropical areas of Africa, South America, Middle-East and Asia and, more recently, it represents an emerging risk in Europe ( Holtfreter *et al.*, 2014).

**Figure 1.**
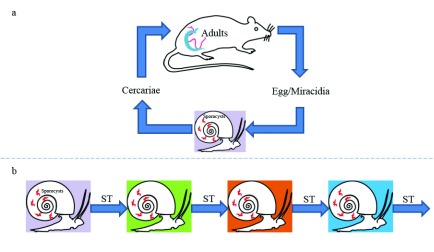
( **a**) The canonical lifecycle of schistosomes includes relatively long periods of development in the two obligatory hosts (a mammal and a snail) and two short-lived, free-swimming larval stages. ( **b**) The reduced lifecycle of schistosomes is exclusive to the snail host, thanks to the technique of sporocyst transfer or transplantation, and bypasses the mammalian host and the free-swimming larval stages. ST: Sporocysts Transfer.

The schistosome lifecycle includes two obligatory hosts ( Figure 1a):

(I) A freshwater snail, in which stages of the parasite, called sporocysts, multiply asexually and produce the mammalian infecting larvae, known as the cercariae, and
(II) A mammalian host in which the parasites (males and females) develop to sexual maturity, pair, mate and produce eggs.

Eggs are excreted from the mammalian host in faeces or urine, and if they reach bodies of freshwater a second free-swimming larval stage, called the miracidium, hatches and infects the snail by active penetration through the integument. After penetration, the miracidium loses the ciliated plates, and develops in less than 18 hours into a mother sporocyst. Daughter sporocysts develop inside the mother sporocyst and eventually escape to invade the snail tissues and organs, mainly the ovotestis and the hepathopancreas. In each daughter sporocyst, germinal cells (similar to stem cells) divide and differentiate to produce either cercariae (cercariogenous sporocysts), or further daughter sporocysts (sporocystogenous sporocysts).

The asexual multiplication of the daughter sporocysts continues throughout the time that the parasite remains resident in the snail ( Théron, 1981). Several generations of daughter sporocysts may occur in the same individual snail, because daughter sporocysts can undergo a re-differentiation into new daughter sporocysts, whether they were sporocystogenous or cercariogenous ( Combes *et al*., 1983). We exploited this reversible developmental plasticity to design a method, called "sporocyst transfer" or "transplantation", generating a direct lifecycle (snail to snail transmission) and bypassing the mammalian host and free-swimming larval stages ( Jourdane *et al.*, 1984) ( Figure 1b). This technique, first developed by Chernin (1966) for *Schistosoma mansoni*, consists of transplanting schistosome sporocysts from one donor snail to recipient snails. It was improved in our laboratory for *S. mansoni* with the snail *Biomphalaria glabrata* as host ( Jourdane, 1977; Jourdane & Théron, 1980; Jourdane & Combes, 1989). We also adapted the procedure to other species of schistosomes like *S. haematobium* and the snail *Planorbarius metidjensis* ( Kéchemir & Combes, 1982), and *S. bovis* in *Bulinus truncatus* ( Jourdane *et al.*, 1984). We describe here, in a video documentary, a detailed and improved method that enables the generation of clonal populations of schistosomes.

The technique of microsurgical transplantation requires a precise protocol and specific skills in snail handling and surgery that can be quite difficult to establish in a laboratory. Therefore, the aim of this publication is to provide for the scientific community a technical video in order to make this technique more broadly available.

## Methods

### Design and development of the video on the transplantation technique

The video explains all the steps, equipment, reagents and manipulations necessary for the technique to be applied by researchers in their own laboratories. It is provided in French ( Video 1), English ( Video 2), Spanish ( Video 3) and Portuguese ( Video 4). There are seven obligatory steps (see protocol in Supplementary File 1). The first step explains the preparation of the donor snail, using an antibiotic solution. The second step explains how to prepare the recipient snails, using an anesthetic solution. The third step provides information on how to prepare the work space and which equipment is needed. The fourth step details the dissection of the donor snail. The fifth step explains how to isolate the sporocyst grafts. The sixth step shows the different manipulations necessary to conduct the transplantation technique properly. The seventh and last step explains how to maintain the recipient snails after the transplantation. The manufacturing process of the two specific tools necessary for the sporocysts transfer, i.e. the microretractor and the glass microsyringe, is presented in Video 5 and Supplementary File 2. Housing, feeding and animal care followed the national ethical standards established in the writ of February 1st, 2013 (NOR: AGRG1238753A). The French Ministère de l’Agriculture et de la Pêche and the French Ministère de l’Education Nationale de la Recherche et de la Technologie provided permit A66040 to the laboratory for animal experiments and certificate to the experimenters (authorization 007083, decree 87–848). All efforts were made to ameliorate any suffering of animals. For details see Supplementary File 1 and the video on sporocyst transfer.

## Discussion

The technical video was designed to make the technique of transplantation of schistosome sporocysts widely available so that it can be used by researchers from different scientific backgrounds, with different levels of research experience and from different countries, including those that speak English, French, Spanish and Portuguese. Many applications of microsurgical transplantation of sporocysts have been highlighted previously ( Jourdane & Combes, 1989) such as:

(i) Selection and maintenance of “pure” and stabilised strains with high compatibility to snails (cloning avoids genetic recombination), resistance to certain antihelmintics, or with specific chronotypes,
(ii) Avoidance of genetic drift (for example in *S. haematobium* where there is a loss of compatibility towards the snail after 2 or 3 passages in the mammal),
(iii) Compensation for the very low success rate of mono-miracidial infections required to produce single sexes of the parasite,
(iv) Maintenance of male and female clones without being forced to identify the sex,
(v) Constitution of genomic libraries,
(vi) Improving the ability to conduct schistosome genetic-based studies, or
(vii) Studying mollusc immunology.

Additional uses of the sporocyst transplantation technique are envisioned based on recent molecular breakthroughs afforded by RNA interference gene knockdown- ( Alrefaei *et al.*, 2011; Mann *et al.*, 2010) and Clustered Regularly Interspaced Short Palindromic Repeats (CRISPR-Cas9) gene knockout- ( Jinek *et al.*, 2012) strategies. Use of the sporocyst transplantation technique in combination with these targeted manipulation strategies will assist the scientific community in functional genomics-based studies of schistosome biology directly in the snail host.

The conditions for successful transplantation are the following: proper anesthesia of the recipient snails; obtaining highest prevalence and post-transplantation survival of the recipient snails; ability of the daughter sporocyst grafts to produce new generation of daughter sporocysts; ability of the new generation of daughter sporocysts to produce enough cercariae (or sporocysts); ability of these cercariae to infect the mammal definitive host (infectivity) and produce eggs with viable and infective miracidia; and the ability to successfully perform several consecutive transplantations.

## Data availability

**Video 1**: Technique de transplantation microchirurgicale de sporocystes de schistosomes.

http://doi.org/10.5281/zenodo.1116997 ( Mouahid *et al.*, 2017a)

**Video 2**: Technique of microsurgical transplantation of schistosomes sporocysts.

http://doi.org/10.5281/zenodo.1117007 ( Mouahid *et al.*, 2017b)

**Video 3**: Técnica de trasplantación microquirúrgica de esporocistos de *Schistosoma*.

http://doi.org/10.5281/zenodo.1117009 ( Mouahid *et al.*, 2017c)

**Video 4**: Técnica microcirúrgica de transplante de esporocistos de esquistossomas.

http://doi.org/10.5281/zenodo.1117011 ( Mouahid *et al.*, 2017d)

**Video 5**: Manufacture of tools for microsurgical transplantation of schistosoma sporocysts.

http://doi.org/10.5281/zenodo.1117017 ( Mouahid *et al.*, 2017e)

License: Creative Commons Attribution 4.0

## Supplementary material

Supplementary File 1: Step-by-step protocol of the technique of microsurgical transplantation of schistosome sporocysts.

Supplementary File 2: Instructions for the manufacture of the tools for sporocyst transfer by microsurgical transplantation.
