# Supplementary material for: Transplantation of schistosome sporocysts between host snails: A video guide
Source: Wellcome Open Res. 2018 Jan 9;3:3. [Version 1] doi: 10.12688/wellcomeopenres.13488.1 (PMC5806052; doi:10.12688/wellcomeopenres.13488.1)
Supplement: Supplementary file 1 [file wellcomeopenres-3-14646-s0000.tgz › 47a98dc3-6c1a-494e-99a9-5ab81b51276f.pdf]

Supplementary File 1

Protocol of the technique of microsurgical transplantation of schistosome sporocysts

Beforehand remark

The technique of microsurgical transplantation of schistosome sporocysts involves the use of living animals (parasites and snails). In order to maintain their physical and physiological integrities, your manipulations must be as gentle and as precise as possible.

Material needed

- ✓ Biological materials: infected snail (donor snail), uninfected snails (recipient snails).
- ✓ Chemical materials: antibiotic solution, anaesthetic, distilled water, PBS, alcohol 70%.
- ✓ Other materials: gloves, glass containers, Petri dishes, strainer, binocular microscope, two pairs of dissecting forceps, a microretractor, a needle, a glass microsyringe.

The seven obligatory steps of the sporocysts transfer

1_ Preparation of the donor snail

- ✓ Prepare the antibiotic solution with well water and nitrofurantoin at 0.1 gram per 1000.
- ✓ Place the donor snail in this solution for 5 hours.

2_Preparation of the recipient snails

- ✓ Prepare the anaesthetic solution by adding 0.89 ml of sodium pentobarbital in 199.11 ml of distilled water. Since we used a sodium pentobarbital stock solution at the

concentration of 182.2 mg of pentobarbital per ml, the final concentration (0.89 ml /199,11 ml d H_2O) corresponds to 0.08g per 100 ml (0.08% in the videos 1 to 4).

- ✓ Wash the recipient snails in a strainer with well water.
- ✓ Place the recipient snails in the anesthetic solution for 5 hours. Thereafter, the recipient snails are asleep, released, and ready to receive a graft. You may need to lightly change the concentration of the anaesthetic solution in order to optimize the snail anaesthesia. However, neither the concentration of the drugs nor your manipulations should put the snails in such a physiological stress (overdose) that the transplantation will not be possible.

3_Preparation of working space and equipment used

- ✓ Disinfect the work space with alcohol (70%).
- ✓ Place the donor snail and the recipient snails around the binocular microscope.
- ✓ On the working space, place:
  - Petri dishes containing previously prepared PBS in which the grafts will be prepared and maintained.
  - Two pairs of dissecting forceps, one for dissecting the shell of the donor mollusc, the other, thinner one, for extracting the grafts.
  - The specific equipment for transplantation: microretractor for the extension of the recipient snail, incision needle and glass microsyringe for the injection of the graft.

4_Dissection of the donor mollusc

- ✓ Place the donor snail in a Petri dish.
- ✓ Dissect the shell following the coils with the dissecting forceps.
- ✓ Delicately detach the columellar muscle.

- ✓ Extract the snail from its shell.
- ✓ Isolate the digestive gland containing the sporocysts and place it in PBS.
- ✓ Remove the integument so that the sporocysts are exposed.

5_Preparation of the grafts

- ✓ Isolate a sample containing 2 to 4 sporocysts with a minimum of the snail tissues (the graft volume is less than 1 cubic millimeter).
- ✓ Prepare three to four grafts.

6_ Transplantation

- ✓ Pre-prime the micro-syringe with PBS.
- ✓ Aspirate the graft.
- ✓ Dry the shell of the recipient snail.
- ✓ Under the binocular microscope, hold the recipient snail between the thumb and the forefinger.
- ✓ Locate the genital pore.
- ✓ Gently place the microretractor in the genital pore.
- ✓ Stretch the mollusc and maintain it in extension.
- ✓ Lay the other end of the micro-retractor and maintain it gently on cotton on the edge of the binocular microscope.
- ✓ Incise the tegument just behind the genital pore with the needle, at the level of the cerebrospinal sinus.
- ✓ Inject the graft with the micro-syringe with a minimum of PBS.
- ✓ Gently remove the micro-retractor.
- ✓ Place the recipient snail in the antibiotic solution for 48 hours.

- ✓ The transplantation or transfer is now complete. About thirty snails can be transplanted from a single donor snail.

7_Maintenance of the recipient snails

- ✓ Place the recipient snails under maintenance conditions and fed them normally.
- ✓ Attention! A cercarial emission occurs during the first week after the transplantation and then stops. The post-transplantation prepatent period is 10 to 20 days longer than during a normal cycle.

How to guarantee the success of the sporocyst transfer?

Performing a sporocyst transfer is a surgical procedure that requires gentle and respectful handling of the snails. This technique is demanding and requires calmness, patience and concentration. The experimenter must handle in a serene setting, without noise and distractions; s/he has to take breaks between transplants. All equipment must be adequate, ready and of good quality. Transplantation requires the manipulation of living material and the snails are likely to respond differently to anaesthesia; if a snail enters its shell during handling, do not hesitate to change it; this is why it is better to anesthetise enough snails in order to have choice (e.g. anaesthetise 45 to transplant into 30).

Good luck!
