# Supplementary material for: Transplantation of schistosome sporocysts between host snails: A video guide
Source: Wellcome Open Res. 2018 Jan 9;3:3. [Version 1] doi: 10.12688/wellcomeopenres.13488.1 (PMC5806052; doi:10.12688/wellcomeopenres.13488.1)
Supplement: Supplementary file 2 [file wellcomeopenres-3-14646-s0001.tgz › 6e12377c-e12b-4e4d-95f4-58d1fc3ffbc5.pdf]

Supplementary File 2

Manufacture of the microretractor

Material

- Bunsen burner
- Glass rod of 3 mm diameter and 20 cm long
- A lighter

Large pair of forceps

Method

1. Start the bunsen burner using the lighter (the flame must be blue)
2. Place the central area of the glass rod in the fire and rotate steadily
3. When the central area of the glass rod becomes soft, remove from the flame and extend it horizontally, consistently and steadfastly to obtain the right diameter to proceed to the step 4
4. Cut off the surplus without getting burned
5. Place the fine end in the flame which, with the heat, will curve naturally. The right size and angle of hook is needed to keep the body of the snail extended during the sporocyst transfer without hurting it.
6. Check under the binocular microscope that the terminal part of the hook is round and ground.
7. Place the other end in the flame, which, under the heat, will also bend naturally at 90 °. While under the flame, flatten the end with a large pair of forceps. This part will serve as a brake and prevent the microretractor from moving during the sporocyst transfer.

Note: Get a good orientation of the hook in relation to the opposite extremity. When the opposite extremity is in a vertical position, the hook must be twisted towards you at an angle of approximately 30 °.

8. Place the microretractor on a support for the cooling phase. The tool is ready.

Manufacture of glass microsyringe

Material

- Bunsen burner
- Pasteur pipette 15 cm long
- A lighter
- Sealing wax
- 1 ml syringe with its needle

Method

1. Start the bunsen burner using the lighter (the flame must be blue)
2. Place the Pasteur pipette in the flame just behind/ posterior to the beginning of the tapered area and turn smoothly and continually.
3. When you feel that the Pasteur pipette is softening, remove from the flame and stretch horizontally, consistently and steadfastly to obtain two parts:
    a. A tapered pipette that can be kept for other activities
    b. A glass microsyringe with a reservoir (similar to bulb pipette or volumetric pipette) in the middle between a long narrow portion at one end (diameter must be sufficiently thin (≈ 0.35 mm)) and the standard pipette tip at the other. This glass microsyringe will be used for the transfer of sporocysts.
    c. Check the quality of the opening of the glass microsyringe under the binocular microscope: it must be sufficiently open and fire-polished to avoid injury to the snails during the sporocyst transfer. If it is not the case, carefully cut the end of the glass microsyringe and pass it through the flame very quickly and check again.
4. Place the shaft of the 1 ml syringe needle into the standard portion of the glass microsyringe.
5. Attach both pieces with the sealing wax

6. Insert the plunger of the syringe and check the effectiveness of the seal and microsyringe by pipetting distilled water.
7. The glass microsyringe is ready.

Note: Avoid any contact between alcohol and the sealing wax.
