# Supplementary material for: Transplantation of schistosome sporocysts between host snails: A video guide
Source: Wellcome Open Res. 2018 Jan 9;3:3. [Version 1] doi: 10.12688/wellcomeopenres.13488.1 (PMC5806052; doi:10.12688/wellcomeopenres.13488.1)
Supplement: Peer review history

# Peer review: Referee response for version 1

**DOI:** 10.21956/wellcomeopenres.14646.r29641

**Published:** 2018-02-07

**Authors:** Christopher J. Bayne

**Affiliations:** 1 Department of Integrative Biology, College of Science, Oregon State University, Corvallis, OR, USA

Mouahid *et al *provide a useful description of sporocyst transplantation. The utility of the protocol is clearly stated and well illustrated in the videos. The comments below are offered as means to improve the precision of the text, and its comprehension.

**TEXT**

 There is no statement as to the success rates achieved in this complex protocol. It would be helpful if, at a minimum, readers were assured that infective cercariae have been routinely obtained from transplanted schistosomes.

 The Legend to Figure 1 can be made more clear by rewording as follows:

 “(b) The cycle can be artificially simplified in a process that avoids the definitive mammalian host: sporocysts are transferred or transplanted between snails, bypassing the mammalian host and the free-swimming larval stages. ST: Sporocyst Transfer.”

 Also, the text below Figure 1 (small font) appears to be lacking one or more initial words. Please check this. Perhaps “Sporocyst transplantation between…”

 the ciliated plates, and **, if the penetrated snail is a susceptible individual,  **develops in less

 hepathopancreas = hepatopancreas

 cercariae ( **in **cercariogenous sporocysts), or further daughter sporocysts ( **in **sporocystogenous sporocysts).

 schistosomes like *S. haematobium = *schistosomes **including** *S. haematobium*

 laboratories. It is provided in French… =  laboratpries. Commentaries are provided in French…

 produce single sexes of the parasite = produce single sex **populations** of the parasite

 forced to identify the sex, = forced to identify the sex (only the adults are sexually dimorphic),

 gene knockout- ( Jinek *et al.*, 2012) strategies. *Delete hyphen.*

 produce new generation = produce **a **new generation

**VIDEO**

 The high quality of the video is noted.

 It is appreciated that modification of the commentary may be, at this stage, challenging. The only version that I have checked is the English one. It is noted that the phrases accompanying the relaxation protocol include this: “Five hours later the snails are asleep, released and ready to…” “released” should be “relaxed”. This is not an essential change.

 I have some concern over the use of PBS. Is this mammalian PBS? Is there a formulation (that can be included in the publication) for a PBS that is physiological for pulmonate snails and their sporocysts? Perhaps the authors use such a formulation. If so, this needs to be clear as some others may assume that mammalian PBS is suitable. And, in the big picture, the techniques work, so a snail PBS may not be essential. But the success rate may be improved if a non-physiological PBS is replaced by a physiological one. The osmotic concentrations of *Biomphalaria* and mammals are markedly different, as described by another of Eli Chernin’s publications ^1^.

I have read this submission. I believe that I have an appropriate level of expertise to confirm that it is of an acceptable scientific standard.

## References

[1] CHERNINE: Observations on hearts explanted in vitro from the snail Australorbis glabratus. *J Parasitol*.1963;49:353-64 14020610

# Peer review: Referee response for version 1

**DOI:** 10.21956/wellcomeopenres.14646.r29642

**Published:** 2018-01-24

**Authors:** Patrick C. Hanington

**Affiliations:** 1 School of Public Health, University of Alberta, Edmonton, AB, Canada

This is an excellent, step-by-step account of the schistosome sporocyst transplantation method developed and refined by this group. The accompanying video and written methodological summary clearly outline each stage of the procedure. Videos are also provided to assist with the development of specific tools to undertake the presented method and generate all required materials.

 The brief written introduction and discussion accurately touch on the importance of this technique and its utility and integration with many tools intended to manipulate the snail/schistosome interface, such as RNAi.

 A fantastic resource for those working in this system. That the videos are made available in multiple languages ensures that many of those working in with these model organisms will be able to access this important resource.

 In the future, it would be great to see accompanying videos explaining any alternations of the technique that are required for implementation of this technique in *Bulinus* sp. snails.

I have read this submission. I believe that I have an appropriate level of expertise to confirm that it is of an acceptable scientific standard.
